# Botulinum Toxin for the Treatment of Tremors

**DOI:** 10.3390/toxins17080401

**Published:** 2025-08-11

**Authors:** Steven Bellows, Joseph Jankovic

**Affiliations:** Parkinson’s Disease Center and Movement Disorders Clinic, Department of Neurology, Baylor College of Medicine, Houston, TX 77030, USA; josephj@bcm.edu

**Keywords:** botulinum, toxin, tremor, essential tremor, Parkinson’s, electromyography, ultrasound

## Abstract

Tremor, an oscillatory movement disorder, is commonly encountered in clinical practice in the setting of a variety of etiologies, such as essential tremor and Parkinson’s disease. Despite its high prevalence, treatment options are somewhat limited. Oral medications are often ineffective or limited by side effects, and other treatments, such as deep brain stimulation, are more invasive and costly. Botulinum toxin (BoNT) injections are a well-established therapy in the treatment of dystonia, but its use in the treatment of tremors has not been fully explored. In this review, we discuss the available randomized controlled trials and open-label evidence for the use of BoNT in various tremor etiologies, as well as its injection techniques. While essential tremor is the most studied condition, other tremor etiologies and tremor types such as Parkinson’s disease, head tremor, voice tremor, proximal tremor, and tremor due to dystonia and multiple sclerosis have been studied as well. Botulinum toxin injections have provided evidence of significant benefit in outcomes in several trials among these indications, but transient weakness remains a common adverse effect. There is a paucity of well-designed trials as many published studies have relatively small cohorts and results are additionally limited by heterogenous outcome measures, dosages, muscle selection techniques and methods of injection.

## 1. Introduction

Tremor is frequently encountered in clinical practice and can be due to many underlying conditions. Upper extremity action tremor is often due to essential tremor (ET), one of the most common movement disorders, with estimated prevalences ranging from 0.32% to 1.33%, and as high as 6% among those older than 60 [1,2]. Parkinson’s disease (PD), another common cause of tremor, has an estimated prevalence of 1% in people over the age of 65 [2,3]. Although rest tremor is present in 68.6% to 87.2% of PD patients [4,5], it is the action (postural and re-emergent) tremor that is most troublesome for patients with PD [6]. Tremor often co-exists with other movement disorders, such as dystonia [7]. Tremor can be seen in a variety of other etiologies, including stroke and multiple sclerosis [8,9].

Although tremor is one of the most troublesome movement disorders, treatment options are limited. In ET, propranolol remains the only FDA-approved medication [1], and along with other drugs, such as primidone and topiramate, medical treatments provide sustained improvement in only 50–60% of patients [10]. Nearly one-third of patients stop therapy due to side effects or insufficient benefit [11], and satisfaction rates with treatment are as low as 11.8% [12]. Levodopa, the primary therapy for PD, is variably effective in treating tremor, and many PD patients exhibit dopamine-resistant tremor [13]. While more interventional treatments, such as deep brain stimulation and focused ultrasound (US), can be effective [14,15], they are associated with potentially more serious adverse events and require notable resource investment.

Botulinum toxin (BoNT) has been evaluated as a potential tremor treatment option for a long time [16], with particular utility as an intermediate option between oral medications and surgical treatment. BoNT injections, tailored to the needs of individual patients, allowing for targeting muscles involved in multiple tremor axes, coupled with a lack of systemic side effects, have added to BoNT’s appeal as a safe and effective treatment modality in patients with troublesome tremors [17]. In this review, we aim to summarize the published data on the use of BoNT in the treatment of tremors, focusing on ET and PD-related tremors. Articles were identified through PubMed using search terms such as “tremor AND toxin” and “tremor AND botulinum”. Articles with full-text availability were prioritized, and additional articles were identified by cross-referencing bibliographies.

## 2. Literature

Despite the high prevalence of tremors, there is a paucity of well-designed therapeutic trials. Botulinum toxin injections have been used as an intermediary therapeutic strategy between medications and surgical intervention, but clinical trials supporting their efficacy are limited by poor design, small sample size, short follow-up and other problems. Furthermore, the published trial results are difficult to compare due to heterogenous tremor etiologies and outcome measures. Many trials include markers of global impressions by clinicians or patients, which may not be the most precise or sensitive method to assess contrasting results. Finally, because of lack of head-to-head comparisons, it is difficult to determine which product, dose, and method of administration are most optimal.

While many studies focused on single etiologies, such as ET, others focused on locations of tremor, such as hand or head, which may be due to several possibly underlying etiologies such as ET or dystonia. For the purposes of this article, we have organized the review by identified target population, rather than by suspected cause of tremor. Studies have been further sub-divided into randomized controlled trials and open-label trials in each section.

### 2.1. Essential Tremor

#### 2.1.1. Randomized Controlled Trials

Only four randomized controlled trials (RCTs) studying BoNT in ET have been reported (Table 1) [18,19,20,21]. The original studies utilized a “fixed-dose” plan with standardized muscle selections and dosages. In 1996, Jankovic et al. randomized 25 ET patients with moderate-to-severe hand tremor to 50 units of onabotulinumtoxinA or placebo [19]. In the active group, 15 units were injected into the flexor carpi ulnaris (FCU) and flexor carpi radialis (FCR) each, while 10 units were injected into the extensor carpi ulnaris (ECU) and extensor carpi radialis (ECR) each. At 4 weeks, if there was no improvement in tremor and no weakness (defined as a 50% decrease in force in the hand or any two digits), a second injection with doubled dosages in each muscle was provided. A variety of tasks were assessed using the Unified Tremor Rating and Assessment (UTRA) ordinal scale. Total UTRA scores were not significantly different, but individual items (such as handwriting and straight line drawing) showed significant improvement at varying time points with BoNT. Postural and kinetic tremor severity were significantly improved at 4 weeks with BoNT, and tremor amplitude by accelerometry was significantly decreased at 4 and 8 weeks as well. Mild-to-moderate finger weakness was reported by 92% (11/12) of BoNT subjects, particularly greater in wrist and finger extensors, but it did not interfere with function.

In 2001, Brin et al. performed another fixed-dose study, in which 133 subjects with ET were injected with either placebo, low-dose (50 units) or high-dose (100 units) onabotulinumtoxinA [18]. The injection protocol was similar to Jankovic et al. [19], with 15 or 30 units in each FCU and FCR, and 10 or 20 units in each ECU and ECR in the low- and high-dose groups, respectively, performed with electromyography (EMG)-guidance. A similar ordinal scale from 0 to 4 was used to assess tremor severity, treatment response, motor tasks, and functional ability. Quality of Life (QoL) and grip strength were also measured. In both dosage groups, postural tremor significantly improved at 6, 12, and 16 weeks, and kinetic tremor improved at 6 weeks. While significant, peak effect was mild. Isolated tremor-assessment tasks improved with either low-dose or high-dose BoNT, with significant improvement in total motor function scores at 6, 12, and 16 weeks with either dosage. There was no significant change in QoL measures, but baseline scores were low already. Grip strength was significantly lower in both dosage groups, and 30% (13/43) of low-dose and 70% (31/45) of high-dose subjects reported subjective hand weakness, including digit extensor weakness. The relatively low impact on functional task improvement was posited to reflect the lesser improvement in kinetic tremor.

Due to mixed outcomes and high prevalences of hand weakness in fixed-dosed protocols (particularly involving extensor muscles), subsequent studies have largely customized muscle and dosage selection in injection plans based on tremor characteristics with substantial reduction in dosage into wrist/finger extensor muscles. In 2018, Mittal et al. evaluated incobotulinumtoxinA versus placebo in 33 patients with ET in a cross-over design with a customized injection plan protocol [21]. Patients were injected using the “Yale technique”, where muscles of interest were identified by evaluating the presence and direction of tremor at the fingers, wrist, and elbow, followed by screening of tremor activity in these muscles by EMG. Final muscle selection and dosage was based on EMG activity and muscle size. Subjects received an average of 9 injections (range 8–14), with an average total of 100 units (range 80–120 units) of incobotulinumtoxinA in the active group. A total of 28 subjects completed the trial, and significant improvement in tremor severity was noted with incobotulinumtoxinA versus placebo at 4 and 8 weeks by multiple tremor severity scales. Patient perspective of “much” improvement (available for 19 subjects) was significantly higher with incobotulinumtoxinA (53% vs. 15%, *p* = 0.031). Mild hand weakness was not significantly different between incobotulinumtoxinA and placebo (six vs. four subjects, *p* = 0.728), but one subject in the incobotulinumtoxinA group withdrew prior to study completion due to troublesome hand weakness, and one incobotulinumtoxinA and three placebo subjects were lost to follow-up for unclear reasons. The lower incidence of clinically meaningful hand weakness (1/28 subjects) was cited as an advantage of customized versus fixed-dose protocols.

Multiple studies have used kinematic analysis for generating injection plans. In this method, tremor is analyzed during several scripted tasks using several sensors placed along the wrist, forearm, elbow, and shoulder, and the results inform muscle selection and dosages for injection. Jog et al. randomized 30 ET subjects to incobotulinumtoxinA or placebo injections using kinematic analysis, and examined tremor severity by Fahn-Tolosa-Marin Tremor Rating Scale (FTM-TRS) and accelerometry and global impression of change at weeks 4 and 8 after a single injection [20]. In the active group, an average dose of 116.3 +/− 53.0 units of incobotulinumtoxinA was injected in an average of 11.7 +/− 3.8 sites, utilizing targeting with local US, EMG, and/or electrical stimulation (ES). In the active group, FCU, FCR, ECR, PT, supinator, pectoralis major, supraspinatus, brachialis, and triceps were injected in >70% of subjects. Significant improvement was noted with incobotulinumtoxinA in FTM part B motor scores and accelerometry amplitude at weeks 4 and 8, and in physician-rated global impression of change at week 4. Total FTM-TRS scores and patient-rated global impression of change were not significantly different. Subjective weakness occurred in 2/19 (10.5%), and grip strength was significantly weaker at week 4 in the incobotulinumtoxinA group compared to placebo.

The ELATE trial is a recently completed, multicenter placebo-controlled RCT examining the use of onabotulinumtoxinA in the treatment of ET. Aiming to enroll 174 participants, subjects were randomized to unilateral upper limb placebo or onabotulinumtoxinA injections in two twelve-week intervals, followed by unilateral or bilateral onabotulinumtoxinA injections in a third injection. The primary outcome at week 18 is change from baseline in the Tremor Disability Scale-Revised (TREDS-R) scale, with several secondary outcomes [23]. The study has been listed as completed on clinicaltrials.gov and is pending a release of results.

#### 2.1.2. Open-Label Trials

Early studies of the use of BoNT in tremor were typically open-label and included mixed etiologies. The use of BoNT for tremor was first examined in 51 patients with mixed etiologies (14 with dystonic, 12 with ET, 22 with combined ET/dystonic tremor, 1 with parkinsonian, 1 with peripherally induced, and 1 with midbrain tremor) [16]. Patients with both head or arm tremor were included, and outcomes were measured on an ordinal scale from 0 (no improvement) to 4 (marked improvement). The average peak effect in the cohort was 3.0, and 35 (67%) patients reported some degree of improvement with BoNT. Transient weakness was noted in 60% of patients treated for hand tremor and 10% of injections for head tremor, and dysphagia in 29% of head tremor injections.

In 1994, Trosch and Pullman reported the results of BoNT injections in 26 patients with upper extremity tremor (12 with PD, 14 with ET) in up to 8 muscles with EMG-guidance [24]. Muscles were surveyed with surface EMG and injected if tremor was confirmed on needle EMG. While only two PD patients and three ET patients had a >50% reduction in quantified tremor amplitude, five PD and five ET patients (38% of total cohort) reported moderate-to-marked improvement subjectively in functional benefit. A subsequent open-label study of BoNT-A in 187 patients included 37 patients with tremor (15 PD, 17 ET, and 5 cerebellar tremor) [25]. BoNT-A was noted to be “generally less effective” for tremor than dystonia or spasticity, with average efficacy ranging from 35.7% to 50.6% in improvement in disability rating and functional rating scores. When measured, quantified tremor amplitude decreased by 13%, 25%, and 19% in PD, ET, and cerebellar tremor, respectively. Excessive weakness was noted in nearly two-thirds of patients with tremor, with most cases involving finger extension weakness after injection of wrist extensors.

In 2018, Niemann and Jankovic reported the “real-world experience” of BoNT injections in a cohort of 91 patients with tremor (53 ET, 31 dystonic, 6 PD, and 1 cerebellar outflow tremor) [26]. Injections were performed largely in the forearm flexors, with 97.8% of limbs injected in these muscles, and 81.3% exclusively so. Injections were performed largely with manual needle placement (MNP) using anatomical landmarks. Using an ordinal scale from 0 (no improvement) to 4 (marked improvement), average peak effect rating after the first injection was 3.2, with 80.2% of patients reporting moderate or marked improvement (3–4 rating). Transient limb weakness occurred in 132/1095 (12.1%) limbs injected.

In another study, 20 ET patients were injected in 1–2 forearm muscles each (mostly FCR and biceps) with abobotulinumtoxinA, utilizing EMG for muscle selection and guidance [27]. Significant improvement was noted in tremor severity, based on both rating scale and accelerometry as well as functional rating scale, and slight weakness in third digit extension developed in 15% of patients. A retrospective review of 50 tremor patients injected with BoNT included 21 patients with ET [28]. Muscle selection was broad, but >75% of patients received injections in pronator teres (PT), supinator, FCU, FCR, ECU, and ECR, with an average of 95.43 +/− 54.07 abobotulinumtoxinA units. Significant improvement at 1 month was noted in activities of daily living (ADL) by the Essential Tremor Embarrassment Assessment (ETEA) and in QoL by the Quality of Life in Essential Tremor Questionnaire (QUEST). Weakness was reported in 18.4% of the entire cohort, without specific data for the ET subgroup. In a similar retrospective review of 15 ET patients treated with EMG-guided injection of onabotulinumtoxinA (<100 units) utilizing the Yale technique, improvements in QoL by QUEST and tremor severity by the FTM-TRS were significant at 1 and 3 months after injection, and 7 (23.3%) subjects developed weakness of unspecified severity.

A series of open-label trials have employed kinematic analysis in generating injection plans. In the first study of ET subjects utilizing this protocol, 24 underwent kinematic analysis, and incobotulinumtoxinA injections were performed at weeks 0, 16, and 32 [29]. Compared to baseline, at week 38, there were significant improvements in tremor severity by FTM-TRS total scores (16.2 +/− 4.6 vs. 9.5 +/− 6.3, *p* < 0.0005) and in QoL by QUEST scores (40.3 +/− 15.8 vs. 27.8 +/− 15.3, *p* = 0.028). Initial injections included an average of 8.8 +/− 2.0 muscles with an average of 169.0 +/− 62.9 incobotulinumtoxinA units, utilizing EMG-guidance. Muscles injected in >75% of patients included FCU, FCR, ECU, ECR, PT, pronator quadratus (PQ), and biceps. Through self-report, mild weakness occurred in two subjects (8.3%), moderate in seven subjects (29.1%), and marked in three subjects (12.5%) after the first injection, and mild weakness in eight subjects (40%) and moderate weakness in four subjects (20%) after the third injection. A subsequent study from the same group showed continued improvement at 96 weeks in mean action tremor score (62.9%, *p* = 0.001) and QUEST scores, and curiously also showed mean action tremor score improvement in the untreated arm (44.4%, *p* = 0.03) [30]. Bilateral upper extremity injections were later shown to have improvement in tremor severity and QoL measures as well [31]. A subsequent retrospective review of patients treated over the past 7 years compared kinematic (43%) versus clinical-expert-derived injection plan (57%), with similar treatment outcomes and discontinuation rates [32].

A recent series of 18 ET patients with severe, medication-resistant tremor evaluated the effectiveness of incobotulinumtoxinA injections with US guidance [33]. Examination, accelerometers, and surface EMG were used to classify patients into supination/pronation (SPP) or flexion/extension (FEP) tremor patterns, with injection of 50 units into the PT in SPP patients and 50 units divided into FCU and FCR in FEP patients. The Essential Tremor Rating Assessment Scale (TETRAS scores) significantly improved by 46.4% (SPP) and 48.2% (FEP), and QUEST scores significantly improved by 65% (SPP) and 62.7% (FEP). Significant improvements were noted in quantified tremor assessments, with mild perceived weakness in two SPP and three FEP patients.

Not all reports of BoNT treatment of tremors have demonstrated substantial benefits. In a retrospective review, 104 ET patients undergoing various treatments completed a battery of self-reported items to gauge satisfaction with treatment [34]. Satisfaction rates ranged from 35% to 57.3%, and dissatisfaction ranged from 9.2% to 37.0%. Significantly higher levels of satisfaction were reported with DBS, but significantly lower levels of satisfaction were reported with BoNT (*p* < 0.05). However, only five patients (4.8%) were receiving BoNT therapy. Furthermore, the analyses captured only one moment in time and many patients were receiving other treatments besides BoNT; additionally, the sample size was very small, and, most importantly, the self-reported data were based on the subjective recollection of response by the patients.

#### 2.1.3. Meta-Analyses

Given the smaller individual cohort sizes, several meta-analyses and reviews have tried to gauge the effectiveness of BoNT for ET. A 2019 review of ET treatments by the Movement Disorder Society deemed BoNT-A as “likely efficacious” for upper limb tremor with concern for dose-dependent weakness, and classified BoNT as “possibly useful” [35]. This review only included the earlier fixed-dose RCTs, as well as a head tremor RCT [18,19,36], and did not include subsequent RCTs with customized dosing plans [21].

A 2020 meta-analysis of BoNT-A in the treatment of tremor including 6 studies with 245 participants showed no difference in clinical tremor scale scores and a significantly higher incidence of adverse effects, including hand weakness [37]. Subgroup analysis suggested a potential benefit for MS-related tremor, but not ET. The authors of this paper, published in the Polish Journal of Neurology and Neurosurgery, however, acknowledged that the analyzed studies were limited by the small number of subjects and marked heterogeneity of tremor etiologies, and, therefore, they “could not make any disease-specific recommendation”. However, another meta-analysis in 2022 including eight trials of BoNT in ET, PD, and MS-related tremor did note overall improvement in hand tremor [38]. Significant reduction in head and hand tremor in ET within 6 weeks of injection was found as well (SMD = −0.58, CI −0.28 to −0.88, I^2^ = 0). Significant benefit was noted with EMG-guidance, but not MNP.

### 2.2. Head Tremor

Head tremor is common, affecting up to 74% of patients with ET [39], and about 1/3 of patients with cervical dystonia have head tremor, although this is probably an underestimate [40,41]. Because oral medications provide limited benefits, BoNT has been increasingly considered as an alternative therapy (Table 2) [42].

#### 2.2.1. Randomized Controlled Trials

The first RCT for head tremor compared onabotulinumtoxinA to placebo in 10 subjects with ET, where 60 units were injected into each splenius capitis (SC) and 40 units into each sternocleidomastoid (SCM) under EMG-guidance [36]. Moderate/marked improvement was noted by clinician rating in 50% of onabotulinumtoxinA subjects versus 10% of placebo subjects. However, there was a high rate of AEs, with neck weakness in 7/10 (70%) and swallowing difficulty in 3/10 (30%) of onabotulinumtoxinA subjects.

The next RCT, published nearly 20 years later, evaluated onabotulinumtoxinA versus placebo in 117 subjects with isolated or essential head tremor, with attempted exclusion of dystonic head tremor [43]. Bilateral SC were injected with 75 units each under EMG-guidance at week 0, and 75 or 100 units at week 12 based on the response from the prior injection. A significantly higher proportion of onabotulinumtoxinA subjects met the primary outcome of ≥2 points on the Clinician Global Impression of Change (CGI-C) scale at week 18 (31% vs. 9%, RR 3.37, *p* = 0.009). Secondary outcomes were generally improved as well, including head tremor severity ratings, quantitative tremor analysis, QUEST scores, and ETEA subscores. AEs were significantly higher with onabotulinumtoxinA compared to placebo (47% vs. 16%, *p* < 0.001). While mostly mild and transient, two AEs required hospitalization, including one occurrence of severe dysphagia resulting in withdrawal from the trial.

#### 2.2.2. Open-Label Trials

While small in number, other trials have been largely consistent in showing that BoNT can provide benefits in patients with head tremor, while carrying a risk of weakness and dysphagia. In Jankovic and Schwartz’s initial description of BoNT for treatment of tremor, 42 of 51 patients had tremors of varying etiologies [16]. Global improvement was noted in 67% of patients with head tremor, but neck weakness occurred in 9.5% and dysphagia in 28.6% of these patients.

One study evaluated abobotulinumtoxinA for the treatment of 43 patients with head tremor (29 dystonic, 14 non-dystonic) [48]. Up to six cervical muscles were selected by visible or palpable tremor and EMG analysis. Patients with lateral oscillatory head tremor received an average of 400 abobotulinumtoxinA units in bilateral SC, and those with anterior–posterior tremor received an average of 500 units in two or more additional muscles. Quantified amplitude of head tremor, Tsui scale scores and pain scores decreased significantly, and 14/14 (100%) non-dystonic and 26/29 (89.7%) dystonic tremor patients noted subjective improvement.

In addition to SC and SCM, other cervical muscles are often involved in head tremor. A retrospective review of 25 patients receiving obliquus capitis inferior (OCI) muscle injections by EMG-guidance, with 23/25 receiving bilateral injections, showed improved pain and patient impression of tremor severity scores [49].

### 2.3. Voice Tremor

Voice tremor is a common manifestation in ET, and is present in 18–30% of ET patients [50]. Essential voice tremor (EVT) is regular and symmetric [51], with horizontal and often vertical laryngeal tremor [52]. BoNT is commonly injected in the thyroarytenoid–lateral cricoarytenoid (TA-LCA) complex, typically percutaneously across the cricothyroid membrane with EMG-guidance. Common side effects include breathiness, hoarseness, and dysphagia, although these often improve within 1 to 2 weeks [10].

No randomized, placebo-controlled trial has been performed for BoNT in voice tremor. However, there are randomized trials assessing dosages, unilateral or bilateral injections, and comparing BoNT to other treatment options.

#### 2.3.1. Comparator Trials

The first randomized trial evaluated unilateral versus bilateral thyroarytenoid (TA) injections for the treatment of voice tremor in a cross-over design [53]. Ten subjects received initially either unilateral 15 units or bilateral 2.5 units of onabotulinumtoxinA and crossed over to the other injection pattern 16 to 18 weeks later. Objective improvement in acoustic measures of tremor severity only occurred in 3/10 bilateral injections and 2/9 unilateral injections, but a subjective reduction in vocal effort (rated by speech–language pathologists) was found in both groups, along with relatively high patient satisfaction scores, and 8 of 10 subjects wished to continue treatment.

Another trial evaluated different bilateral vocal cord dosages in treating tremor [54]. Thirteen subjects with voice tremor without spasmodic dysphonia were randomized to 1.25, 2.5, or 3.75 units of BoNT-A in bilateral vocal cords. When combining all dosage groups, significant improvement was noted in several measures as compared to baseline, including patient-rated severity scales, functional disability ratings, clinician ratings of videotaped speech segments, and selected acoustic measures. While the cohort was too small for definitive assessment, there was a suggestion of dose-dependent improvement in outcomes. AEs were common, including 11/13 with breathiness and 3/13 with dysphagia at week 2, with all but 1 resolved by week 6.

Justicz et al. evaluated propranolol versus BoNT (the type was not mentioned) in 18 patients with EVT [55]. After baseline voice recordings, patients were treated with propranolol 10–30 mg three times per day for 2 weeks. After a second evaluation, propranolol was stopped and patients received BoNT injections in the thyroarytenoid/lateral cricoarytenoid complex (TA/LCA), with 2/18 with unilateral and 16/18 with bilateral injections and an average 3.18 unit dose. BoNT, but not propranolol, showed significant improvement in Voice-Related Quality of Life (VRQOL) scores, but no significant change in QUEST scores was found, and there was no significant change in expert-rated scores of audio samples.

In a similar trial, 15 patients with essential or dystonic vocal tremor were randomly assigned to receive abobotulinumtoxinA or propranolol, followed by the other treatment in a cross-over design [56]. BoNT treatment consisted of 15 units of abobotulinumtoxinA injected unilaterally into the left TA, while propranolol was dosed at 40 mg twice per day. In EVT, no difference in perceptual or acoustic analyses was noted before or after treatments, or between the two treatment arms. In dystonic voice tremor, overall levels of vocal changes were not significantly different, but vocal instability and variability in fundamental frequency were significantly improved with BoNT compared to propranolol.

A cross-over trial which enrolled seven patients with EVT compared BoNT against injection augmentation (IA), where filler material is injected into the vocal folds to improve fold closure. All patients received BoNT (five bilateral, two unilateral adductor muscle complex injections; average 1.26 units of onabotulinumtoxinA) followed by IA. No significant difference between treatments were noted on videostroboscopy rating, acoustic/aerodynamic rating, audio–perceptual assessment, or self-assessment measures, except for perceptual ratings of loudness, which were significantly decreased with BoNT and increased with IA.

#### 2.3.2. Open-Label Trials

In an open-label trial of BoNT injections (mostly in TA) in 15 patients with voice tremor, 10/15 (67%) noted subjective benefit, and there was a significant reduction in voice tremor by perceptual evaluations [57]. In a trial involving 16 patients with EVT, injection plans were adjusted based on laryngeal tremor characteristics: those with horizontal laryngeal tremor were injected in TA muscles, while vertical laryngeal tremor was treated with strap muscle injections [58]. Combined tremor types received a second staged injection after 2 weeks if the first had insufficient benefit. While 94% of patients had horizontal glottic tremor, 75% had combined tremor patterns as well, and 93.8% received TA injections and 43.8% received strap muscle injections. Average TA dosage was 1 unit, and average strap muscle dosage was 3.2 units. All patients reported benefits, but objective measures were not reported. Mild, transient hoarseness developed in 8 of 15 patients with TA injections. In a retrospective review of 319 patients receiving BoNT for spasmodic dysphonia (SD) or EVT, bilateral injections with equivalent dosages had the longest duration of improved voice symptoms, but unilateral injections had significantly reduced voice breathiness and dysphagia, with 92% of patients eventually switching to a unilateral regimen [59].

#### 2.3.3. Reviews

A scoping review of evidence-based treatment for EVT included analysis of BoNT among other treatment modalities [60]. Very little high-quality evidence was found for any treatment modality; primarily, BoNT was found to have similar responses between unilateral and bilateral injection plans, and it did not display superiority compared to injection augmentation.

### 2.4. Parkinson’s Disease

Despite the high prevalence of PD and PD-related tremors, many of which are resistant to levodopa [13], there is a relative paucity of data for BoNT in the treatment of PD-related tremors. Furthermore, many studies fail to differentiate the various types of tremors in PD, such as rest tremor, postural tremor, and re-emergent tremor, that can be quite troublesome, ranging from socially embarrassing to physically disabling. Overall, tremor improvement appears to be more modest in PD-related tremors than in ET [61].

#### 2.4.1. Randomized Controlled Trial

A single RCT has been performed for BoNT in PD tremor (Table 1), where 30 patients were randomized to incobotulinumtoxinA or placebo, followed by cross-over to the other treatment modality [22]. Injection plans were created using the Yale technique of EMG screening, with an average of 100 units injected over nine injection sites. Common muscles (>75% of patients) included lumbricals, FDS, FCU, FCR, pronator, biceps, and triceps. Significant improvement was noted in rest and action tremor by Unified Parkinson’s Disease Rating Scale (UPDRS) rating and Patient Global Impression of Change (PGI-C) but not QoL ratings at 8 weeks with BoNT compared to placebo. While there was no significant increase in hand-weakness-by-grip-strength assessment, three BoNT patients perceived subtle weakness, and two BoNT and one placebo patient perceived moderate-to-severe hand weakness. While satisfaction rates were higher with BoNT (50% vs. 26%), only one-third reported substantial improvement in their tremor.

#### 2.4.2. Open-Label Trials

Open-label data largely come from PD patients included in mixed tremor etiology cohorts. Among 12 PD patients included in the Trosch and Pullman study, 5/12 had a greater than 50% reduction in tremor, and 5/12 had moderate/marked functional improvement, despite an average 15% amplitude reduction among all 12 patients [24]. The later study by Pullman et al. included 15 PD patients, but only 2/15 demonstrated greater than 50% reduction in tremor and satisfactory functional improvement [25]. The more recent 91-patient cohort from Niemann and Jankovic included 6 PD patients receiving a mean of 47.9 +/− 11.5 units in FCU and FCR with moderate/marked benefit reported in 80.2% [26].

Kinematic analysis has been used not only in ET but also in PD patients. A pilot study of seven PD patients were injected with incobotulinumtoxinA using clinical and kinematic assessments, with significant kinematic improvement at 2 and 3 months and UPDRS rest tremor item scores at 1, 2, and 3 months [62]. A follow-up study of 28 PD patients utilized kinematic analysis in generating injection plans, with incobotulinumtoxinA injections at 0, 16, and 32 weeks [63]. By the third treatment, the most frequently injected muscles included FCR, ECR, PT, PQ, and supinator, with average total dose of 186.7 +/− 79.5 units. UPDRS rest tremor scores were significantly lower from baseline (2.7 +/− 0.6) to week 16 (2.0 +/− 0.8, *p* = 0.006) and week 2 (2.1 +/− 0.7, *p* = 0.014). FTM part A scores (tremor severity) were significantly reduced only at week 6. Many accelerometry measures, particularly measured at the hand, were significantly decreased throughout the study. Slight/mild weakness in third finger extension developed in 12/21 (57%) patients.

Another study from the same group enrolled 47 PD patients utilizing kinematic analysis for injection plans [64]. Injection totals ranged from 65 to 390 units by the fourth injection. BoNT resulted in significant improvement in tremor amplitude (41.6%) as well as UPDRS rest tremor score, QoL measures, and arm function (by FTM part C) scores. While mild weakness by manual muscle testing occurred in only 1.9% of assessments, some degree of weakness was reported in 36/45 (80.6%) and moderate weakness in 17/47 (36.2%), with 6 participants withdrawing due to functional impairment from perceived weakness.

A single series examined BoNT for jaw tremor in three patients, where masseters were injected with 30–100 units of abobotulinumtoxinA, resulting in improvement in tremor by clinician impression [65].

A systematic review included one RCT and nine OL studies for a total of 131 participants with varied outcome groups [66]. Average reduction in UPDRS rest tremor item was 1.8 +/− 2.5, and in the UDPRS action tremor item, it was 1.2 +/− 3.8, but a high prevalence of dose-dependent side effects, including weakness, was noted. Common muscles included ECR, ECU, FCR, FCU, PT, biceps, and triceps. Despite typically greater degrees of metacarpophalangeal (MCP) involvement in PD tremor, lumbricals were often not included. Gao et al. performed a meta-analysis of BoNT for various PD symptoms, showing overall effectiveness in BoNT for treating tremor [67]. However, only two studies were able to be included for the tremor analysis, including one with seven patients that had been primarily assessing improvement in pain [68].

### 2.5. Other Tremor Types

#### 2.5.1. Tremor Due to Multiple Sclerosis

Tremor is present in up to 26% to 58% of MS patients, and can be strongly associated with impairment and disability [69]. Treatment is often difficult, and evidence for oral medication options is limited. BoNT has been evaluated for MS tremor in several trials (Table 2).

##### Randomized Controlled Trials

In a cross-over RCT comparing onabotulinumtoxinA to placebo, 33 upper limbs (UL) were injected in 23 patients at baseline and 12 weeks [44]. Enrolled patients had relapsing-remitting MS (RRMS) or secondary progressive MS (SPMS) with disabling arm tremor (mostly moderate by Bain TRS) and stopped oral medications one week prior to baseline injection. Agonist/antagonist muscle pairs were chosen after clinical tremor assessment, and injections were performed under EMG-guidance with ES if needed. A mean dosage of 83 units was injected per limb, and limbs were randomized individually to BoNT or placebo, with cross-over to the alternate group at the second injection. Significant improvement was noted in Bain TRS scores for tremor severity, writing, and spiral drawing at 6 and 12 weeks. QUEST scores did not significantly change from baseline. Mild to moderate weakness was significantly higher with BoNT (42.2% vs. 6.1%, *p* = 0.0005) but resolved in all patients within 2 weeks.

Boonstra et al. aimed to evaluate the effect of BoNT on brain activation patterns in MS patients [45]. Forty-three MS patients with tremor were randomized to onabotulinumtoxinA or placebo, and assessed with Bain TRS for severity, handwriting, and spiral drawing at baseline, 6, and 12 weeks, and underwent functional MRI (fMRI) at baseline and 6 weeks. An average of 67.3 +/− 18.6 units were injected in BoNT patients under EMG-guidance. Significant improvement occurred in handwriting tremor at 6 and 12 weeks, and in tremor severity at 12 weeks compared to baseline with BoNT, correlated with a change in ipsilateral inferior parietal cortex activation. However, there was no significant change in tremor between BoNT and placebo. A significant decrease in muscle strength occurred at 6 weeks compared to baseline, but this recovered close to baseline by 12 weeks.

##### Open-Label Trials

In one of the first trials examining BoNT for MS tremor, five MS patients with cerebellar tremor were injected with 40 units of BoNT-A in flexor and extensor compartments [70]. An additional 100 units were injected in two patients after two months. No significant improvement in intention tremor was noted at any time point, and weakness was dose-limiting.

In a retrospective cohort, eight patients with SPMS received onabotulinumtoxinA for severe arm tremor [71]. Up to 100 units were injected in each patient, with all plans including proximal muscles and smaller injections in wrist flexors under EMG-guidance. Significant improvement was found in FTM-TRS part A tremor scores, part B hand function scores, ADL scores, and total scores. Maximal amplitudes reduced from an average 1350 to 725 microvolts. No patients developed weakness.

#### 2.5.2. Proximal Tremor

Other etiologies besides MS may generate Holmes tremor (HT) or other proximal tremors, including dystonia and ET. These proximal tremors remain challenging to treat with oral medications alone.

##### Randomized Control Trial

One recent double-blind RCT randomized 20 patients with proximal tremor (10 dystonic, 7 ET, 1 HT, 1 PD tremor) to incobotulinumtoxinA or placebo, with cross-over at the second injection at 4 months (Table 2) [46]. Injection plans were determined by clinical assessment, and an average of 180 +/− 60 units was injected with EMG-guidance. There was no significant difference in the primary outcome of Goal Attainment Rating Scale (GARS), but there was a significant reduction in TETRAS (−1.05, *p* = 0.03) and FTM-TRS total scores (−6.84, *p* = 0.022). However, there was no significant change in patient-related outcomes, including QUEST and Bain and Findley ADL scores. Mild and severe weakness occurred in 7/20 (35%) and 1/20 (5%) of patients, respectively.

#### 2.5.3. Dystonic Tremor

Despite the large amount of data regarding BoNT in treatment of dystonia, there are relatively few studies examining BoNT specifically for dystonic tremor (Table 2). Benefit has been noted for dystonic subgroups in head tremor [48] and voice tremor [56] as described above, and head tremor benefit has been noted in older cohorts of cervical dystonia patients [72,73,74].

##### Randomized Controlled Trial

There is a single RCT examining onabotulinumtoxinA versus placebo in treatment of dystonic hand tremor in 30 patients [47]. Postural tremor was present in 23/30 (76.7%), kinetic/intention tremor in 20/30 (66.7%) and rest tremor in 12/30 (40.0%). An average of 63 units was injected across an average of six muscles under EMG-guidance. No significant difference in FTM-TRS scores was found at 6 or 12 weeks, but significantly more patients reported improvement with BoNT per the PGI-C without significant differences in subjective or objective assessments of hand weakness between groups.

##### Open-Label Trials

Jankovic and Schwartz’s initial report on BoNT in tremor included 14 patients with dystonic tremor and 22 patients with combined dystonic and ET [16]. Using an ordinal scale of tremor improvement (3 = moderate improvement, 4 = marked improvement), dystonic tremor had an average 3.5 +/− 0.6 benefit, while combined tremor had an average 3.0 +/− 1.4 benefit. Niemann and Jankovic’s subsequent 2018 mixed-tremor cohort included 37 patients with dystonic tremor who received an average of 77.3 units of onabotulinumtoxinA, mostly in FCU and FCR, but 11 of those 37 patients received injections in the abductor pollicis brevis (APB) as well [26]. As noted, moderate/marked benefit was noted in 80.2% of the entire cohort at their last injection, but specific dystonia subgroup results were not available.

In a retrospective review of treatment courses of 47 patients with dystonic tremor, 31 had received BoNT [75]. Patient-rated improvement in symptoms was noted in 92% of BoNT patients versus 39% of those receiving oral medications. While 23/31 patients receiving oral therapy stopped due to side effects or lack of improvement, weakness was present in 7/31 BoNT patients, resulting in 2/31 patients stopping therapy.

#### 2.5.4. Task-Specific Tremor

Trials for task-specific tremors are difficult to design because each patient appears to have a unique tremor, triggered or exacerbated by a specific activity or position; hence, most studies are open-label and small. In one review of treatments for a series of patients with musician’s tremor, 5 patients had received BoNT and were injected with 10–117.5 units among 2–6 muscles, with 4/5 (80%) of patients reporting improvement in tremor [76]. In a trial of BoNT for primary writing tremor (PWT), four patients were injected with 10–12.5 units per muscle across 1–3 muscles, with moderate/marked improvement in all patients [77]. Mirror dystonia was present in 3/4 patients, suggestive of dystonic etiology.

#### 2.5.5. Functional Tremor

One RCT examined abobotulinumtoxinA versus placebo in 48 patients with functional tremor, with a primary outcome of dichotomized video-rated Clinician Global Impression of Improvement (CGI-I), defined as improved versus no change or worsening [78]. While there was no significant difference between groups, a large portion of both BoNT (64%) and placebo (56.5%) groups met this primary outcome. A similar dichotomized patient impression of improvement occurred in 48% of BoNT and 52.2% of placebo patients. The average initial dosage in the BoNT arm was 240 abobotulinumtoxinA units, but in contrast to other studies, injection plans frequently included trunk or leg muscles. Weakness developed in 24% of BoNT and 17.4% of placebo patients. After an open-label extension, 35/43 (81.4%) of patients had improved compared to baseline. While superiority of BoNT could not be demonstrated, the study did recapitulate large placebo effects that have been seen in other functional neurologic disorder treatment trials [79].

## 3. Targeting Modalities and Techniques

BoNT injections for indications such as cervical dystonia may often be performed by MNP using anatomical landmarks without additional guidance. However, the potential benefits from guidance techniques have been examined. Low rates of targeting accuracy have been reported with MNP alone [80], including upper limb accuracy rates of 37% in dystonia [81] and 39% to 63% in stroke [82]. It is important to emphasize, however, that the target muscles must be identified via a skilled examination aiming to identify the primary agonist muscles involved in the abnormal involuntary movement. EMG records muscle activity but this does not mean that the muscle activity corresponds to the generation of abnormal movement or position, rather than activity generated by compensatory (antagonist) muscle. The latter should clearly not be targeted for BoNT injection. The full extent of the phenomenology of the movement disorder can be better appreciated when examining for overflow and “mirror” movements and postures [83]. EMG can assess tremor burst activity, and studies using the Yale technique have based muscle selection for upper limb tremor injections on EMG activity [21,84]. US allows for direct visualization of the muscles, but it is the expert examination that determines which of these muscles are involved in abnormal movement or posture, and thus, be targeted with BoNT. This technique, however, may help in avoiding nearby structures and has been used widely by other specialties such as anesthesiology [85]. More precise localization has been noted to reduce AEs, such as dysphagia in cervical dystonia [86,87]. Combining US and EMG may yield additional benefits with the confirmation of both location and tremor activity of target muscles.

Some trials of BoNT in tremors have used MNP without guidance [16,19,26]. However, subsequent RCTs and open-label trials have largely used additional guidance tools, most predominantly EMG [18,20,21,22,29,36,43,44,46,47]. While there are isolated trials evaluating the use of guidance and comparing modalities for BoNT in tremor, more data have been published for dystonia indications.

A review of targeting modalities evaluated 10 total studies, including 7 RCTs, 9 prospective trials, and 6 studies classified as level 1 evidence [80]. EMG was superior to MNP in two CD and one spasticity study [88,89,90]. ES has demonstrated more benefit than MNP on upper limb spasticity in adults [91] and some spasticity and function measures in children with cerebral palsy [92]. US has shown improvement on several spasticity measures in adults and children [91,93,94]. Another review of nine studies similarly noted level 1 evidence that US, EMG, and ES were superior to MNP in improving spasticity outcomes [95].

A more recent case series used US guidance for BoNT injections for tremor [96]. In 18 ET patients receiving incobotulinumtoxinA based on US guidance, patients demonstrated significant improvement in tremor severity scores and QoL, with mild digit weakness in 5/18 (27.8%) patients [33].

Several reviews have reported the benefit of targeting modalities over MNP alone [80,91,95,97]. However, the American Academy of Neurology 2016 practice guideline did not favor MNP or any targeting modality [98], and a 2015 consensus statement on BoNT in CD noted that EMG or US would be required for targeting deeper muscles [99].

## 4. Discussion

Despite the high prevalence of tremor and the common use of BoNT in other indications, the number of studies examining BoNT in tremor remains small, with a particular lack of RCTs. Larger, well-designed randomized controlled trials are needed in all tremor etiologies to better understand the potential benefits of BoNT. ELATE, a multicenter phase 2b RCT examining the use of onabotulinumtoxinA versus placebo in the treatment of ET, has completed enrollment and is pending release of topline results [23].

Although some studies have reported better outcomes using US and/or EMG targeting, the relative benefits are quite small compared to MNP, particularly when the latter is performed by a skilled and experienced clinician, and they should be weighed against increased procedure time, more and longer discomfort, and increased cost associated with these techniques. These techniques may be appropriate when better outcomes or fewer AEs are desired.

While fixed-dose protocols have largely been supplanted by customized injections, there is still an unmet need for better designs and tremor and assessments. Given the frequency of extensor weakness in trials, some advocate minimizing or avoiding injections in extensor muscles [10,84]. Wrist flexion/extension has long been a primary tremor axis targeted for injections [16,18,19], with some series nearly solely focusing on wrist flexor injections [26]. However, other axes, particularly forearm pronation/supination, have been increasingly identified in ET [100], and subsequently specifically targeted in some injection protocols [20,21,46]. Many studies have utilized clinical assessment as the primary method of generating injection plans, while others have advocated for EMG screening (such as the Yale technique) or kinematic analysis using accelerometers across multiple joints [20,21]. The Yale technique may use overall less BoNT, and kinematic analysis may have similar results as to expert-derived injection plans, but comparison data remains limited [32,101]. Computer vision analysis of tremors is being increasingly studied [102,103], and may prove useful in the analysis of tremor characteristics for the generation of injection plans.

Several other limitations exist beyond the paucity of well-designed clinical trials and relatively small sample sizes. Study methodologies utilize different toxins, injection techniques, muscle selection, and targeting guidance. A variety of outcome scales have been utilized, limiting the comparison of results. Trials targeting manifestations of tremor, such as head tremor, may inadvertently include different underlying etiologies, such as ET or cervical dystonia. Head-to-head comparisons of different products, injection techniques or selection patterns are lacking. All these confounding factors contribute to limited generalizability of trial results.

## 5. Conclusions

Tremor can arise from a variety of etiologies and remains a common and impairing problem for many patients. Oral therapy often has partial or limited benefit, and surgical treatments may be associated with potentially more serious AEs. BoNT may serve as a useful intermediate therapy for a variety of tremor etiologies and further benefits from a paucity of generalized side effects. Although RCTs and multiple smaller open-label trials have shown the benefit of BoNT in tremor, particularly in ET, more data are needed to validate its effect and determine ideal administration protocols.

## Figures and Tables

**Table 1:** Randomized Controlled Trials for Botulinum Toxin in Essential Tremor and Parkinson’s Disease.

Trial	Dx	N	Frequent Muscles Injected (>67%)	Toxin	Total Mean Dosage (Units)	Significant Improvement	No Significant Improvement	Adverse Effects
Jankovic et al., 1996 [19]	ET	25	FCU, FCR, ECU, ECR	ONA	50	Handwriting, line drawing Postural/kinetic tremor at 4–8 weeks Tremor amplitude (accelerometry)	UTRA total scores	Mild/moderate weakness: 11/12 (92%)
Brin et al., 2001 [18]	ET	133	FCU, FCR, ECU, ECR	ONA	50/100	Postural tremor Kinetic tremor (6 weeks) Total motor function scores	QoL scores	Weakness (low-dose): 13/43 (30%) Weakness (high-dose): (31/45): 70%
Mittal et al., 2018 [21]	ET	28	Lumbricals, FDS, FCU, FCR, ED, ECR, PT, biceps (utilizing EMG screening by Yale technique)	INCO	100	Tremor severity (FTM-TRS) NIHCGC Patient-assessed “much improvement”		Mild weakness: 6/28 (25%) Severe weakness: 1/28 (3.6%)
Jog et al., 2020 [20]	ET	30	PQ, PT, FCU, FCR, ECR, supinator, brachialis, triceps, pec major, supraspinatus (utilizing kinematic analysis)	INCO	116	FTM part B motor scores Tremor amplitude (accelerometry) Physician impression of change (week4)	Total FTM-TRS scores Patient impression of change	Weakness: 2/19 (10.5%)
Mittal et al., 2017 [22]	PD	30	Lumbricals, FDS, FCU, FCR, PT, biceps, triceps (utilizing EMG screening by Yale technique)	INCO	100	Rest, action tremor (UPDRS items) PGI-C	QoL ratings	Subtle weakness: 3/15 (20%) Moderate-to-severe weakness: 2/15 (13.3%)

Dx = diagnosis, ET = essential tremor, PD = Parkinson’s disease, FCU = flexor carpi ulnaris, FCR = flexor carpi radialis, ECU = extensor carpi ulnaris, ECR = extensor carpi radialis, FDS = flexor digitorum superficialis, ED = extensor digitorum, PT = pronator teres, PQ = pronator quadratus, ONA = onabotulinumtoxinA, INCO = incobotulinumtoxinA, FTM-TRS = Fahn-Tolosa-Marin Tremor Rating Scale, NIHGCG = National Institutes of Health Collaborative Genetic Criteria tremor score severity, UPDRS = Unified Parkinson’s Disease Rating Scale, PGI-C = patient global impression of change, UTRA = Unified Tremor Rating and Assessment, QoL = Quality of Life, AE = adverse effect.

**Table 2:** Randomized Controlled Trials for Botulinum Toxin in Other Tremor Types.

Trial	Dx	N	Most Frequent Muscles Injected	Toxin	Total Mean Dosage (Units)	Significant Improvement	No Significant Improvement	Adverse Effects
Pahwa et al., 1995 [36]	HT	10	SC	ONA	200		Moderate/marked improvement by clinician rating in 50% vs. 10% (BoNT vs. placebo)	Neck weakness: 7/10 (70%) Swallowing difficulty: 3/10 (30%)
Marques et al., 2023 [43]	HT	117	SC	ONA	75/100	≥2 on CGI-C (week 18) in 31% vs. 9% Median TRS subscores at several weeks Tremor amplitude QUEST (weeks 12, 18) ETEA subscores	Total median TRS score (except week 24) QUEST (weeks 6, 24)	Pain (34%) Weakness (15%) Dysphagia (16%) Cervical stiffness (10%)
Van Der Walt et al., 2012 [44]	MS	23 (33 limbs)	n/a	ONA	83	Bain TRS (tremor severity, writing, spiral drawing) at 6/12 weeks	QUEST	Weakness: 14/33 limbs (42.2%)
Boonstra et al., 2020 [45]	MS	43	Biceps, pronator teres, FCU, FCR	ONA	67.3	Bain TRS handwriting (6, 12 weeks) vs. BL Bain TRS tremor severity (12 weeks) vs. BL	No change in tremor scores vs. placebo	“Significant decrease in muscle strength” at week 6
Nagaratnam et al., 2025 [46]	PT	20	n/a	INCO	180	TETRAS total score FTM-TRS total score	GARS QUEST Bain and Findley ADL scores	Mild weakness: 7/20 (35%) Severe weakness: 1/20 (5%)
Rajan et al., 2021 [47]	DT	30	FCU, FCR, ECU	ONA	63	PGI-C	FTM-TRS	No difference vs. placebo

Dx = diagnosis, HT = head tremor, MS = multiple sclerosis, PT = proximal tremor, DT = dystonic tremor, SC = splenius capitis, FCU = flexor carpi ulnaris, FCR = flexor carpi radialis, ECU = extensor carpi ulnaris, ONA = onabotulinumtoxinA, INCO = incobotulinumtoxinA, CGI-C = Clinician Global Impression Of Change, TRS = Tremor Rating Scale, QUEST = Quality of Life in Essential Tremor Questionnaire, ETEA = Essential Tremor Embarrassment Assessment, TETRAS = The Essential Tremor Rating Assessment Scale, FTM-TRS = Fahn-Tolosa-Marin Tremor Rating Scale, GARS = Goal Attainment Rating Scale, ADL = activities of daily Living, AE = adverse effect.

## Data Availability

No new data were created or analyzed in this study. Data sharing is not applicable to this article.

## References

[1] Okelberry T., Lyons K.E., Pahwa R. (2024). Updates in Essential Tremor. Park. Relat. Disord..

[2] O’Shea S.A., Shih L.C. (2023). Global Epidemiology of Movement Disorders: Rare or Underdiagnosed?. Semin. Neurol..

[3] Willis A.W., Roberts E., Beck J.C., Fiske B., Ross W., Savica R., Van Den Eeden S.K., Tanner C.M., Marras C. (2022). Incidence of Parkinson Disease in North America. Npj Park. Dis..

[4] Gupta D., Kuo S.-H. (2018). Prevalence and Patterns of Rest, Postural and Action Tremor in Drug-Naïve Parkinson’s Disease (P2.066). Neurology.

[5] Pasquini J., Deuschl G., Pecori A., Salvadori S., Ceravolo R., Pavese N. (2023). The Clinical Profile of Tremor in Parkinson’s Disease. Mov. Disord. Clin. Pract..

[6] Jankovic J. (2016). How Do I Examine for Re-Emergent Tremor?. Mov. Disord. Clin. Pract..

[7] Albanese A., Bhatia K.P., Fung V.S.C., Hallett M., Jankovic J., Klein C., Krauss J.K., Lang A.E., Mink J.W., Pandey S. (2025). Definition and Classification of Dystonia. Mov. Disord. Off. J. Mov. Disord. Soc..

[8] Rodriguez-Porcel F., Sarva H., Joutsa J., Falup-Pecurariu C., Shukla A.W., Mehanna R., Śmiłowska K., Lanza G., Filipović S.R., Shalash A. (2024). Current Opinions and Practices in Post-Stroke Movement Disorders: Survey of Movement Disorders Society Members. J. Neurol. Sci..

[9] Vaheb S., Dehghani Firouzabadi D., Ghoshouni H., Yazdan Panah M., Shaygannejad V., Mirmosayyeb O. (2025). Frequency of Tremor in People with Multiple Sclerosis: A Systematic Review and Meta-Analysis. Clin. Park. Relat. Disord..

[10] Mittal S.O., Lenka A., Jankovic J. (2019). Botulinum Toxin for the Treatment of Tremor. Park. Relat. Disord..

[11] Shah C., Jackson G.R., Sarwar A.I., Mandava P., Jamal F. (2022). Treatment Patterns in Essential Tremor: A Retrospective Analysis. Tremor Hyperkinetic Mov. N. Y..

[12] Louis E.D., Rohl B., Rice C. (2015). Defining the Treatment Gap: What Essential Tremor Patients Want That They Are Not Getting. Tremor Hyperkinetic Mov..

[13] Zach H., Dirkx M.F., Roth D., Pasman J.W., Bloem B.R., Helmich R.C. (2020). Dopamine-Responsive and Dopamine-Resistant Resting Tremor in Parkinson Disease. Neurology.

[14] Mukherjee A., Pandey S. (2025). Current and Emerging Therapeutic Targets for Essential Tremor: A Critical Appraisal of Recent Preclinical and Clinical Studies. Mov. Disord. Clin. Pract..

[15] Wong J.K., Viswanathan V.T., Nozile-Firth K.S., Eisinger R.S., Leone E.L., Desai A.M., Foote K.D., Ramirez-Zamora A., Okun M.S., Wagle Shukla A. (2020). STN Versus GPi Deep Brain Stimulation for Action and Rest Tremor in Parkinson’s Disease. Front. Hum. Neurosci..

[16] Jankovic J., Schwartz K. (1991). Botulinum Toxin Treatment of Tremors. Neurology.

[17] Gulati K., Pandey S. (2025). Botulinum Toxin for Essential Tremor. Toxicon.

[18] Brin M.F., Lyons K.E., Doucette J., Adler C.H., Caviness J.N., Comella C.L., Dubinsky R.M., Friedman J.H., Manyam B.V., Matsumoto J.Y. (2001). A Randomized, Double Masked, Controlled Trial of Botulinum Toxin Type A in Essential Hand Tremor. Neurology.

[19] Jankovic J., Schwartz K., Clemence W., Aswad A., Mordaunt J. (1996). A Randomized, Double-Blind, Placebo-Controlled Study to Evaluate Botulinum Toxin Type A in Essential Hand Tremor. Mov. Disord..

[20] Jog M., Lee J., Scheschonka A., Chen R., Ismail F., Boulias C., Hobson D., King D., Althaus M., Simon O. (2020). Tolerability and Efficacy of Customized IncobotulinumtoxinA Injections for Essential Tremor: A Randomized, Double-Blind, Placebo-Controlled Study. Toxins.

[21] Mittal S.O., Machado D., Richardson D., Dubey D., Jabbari B. (2018). Botulinum Toxin in Essential Hand Tremor—A Randomized Double-Blind Placebo-Controlled Study with Customized Injection Approach. Park. Relat. Disord..

[22] Mittal S.O., Machado D., Richardson D., Dubey D., Jabbari B. (2017). Botulinum Toxin in Parkinson Disease Tremor: A Randomized, Double-Blind, Placebo-Controlled Study With a Customized Injection Approach. Mayo Clin. Proc..

[23] Barbano R., Simpson D., Patterson K., Alibhai N., James L. (2024). A Phase 2b Multicenter, Randomized, Double-Blind, Placebo-Controlled Study of OnabotulinumtoxinA for the Treatment of Upper Limb Essential Tremor: ELATE Trial in Progress. Toxicon.

[24] Trosch R.M., Pullman S.L. (1994). Botulinum toxin a injections for the treatment of hand tremors. Mov. Disord..

[25] Pullman S.L., Greene P., Fahn S., Pedersen S.F. (1996). Approach to the Treatment of Limb Disorders with Botulinum Toxin A. Experience with 187 Patients. Arch. Neurol..

[26] Niemann N., Jankovic J. (2018). Botulinum Toxin for the Treatment of Hand Tremor. Toxins.

[27] Pacchetti C., Mancini F., Bulgheroni M., Zangaglia R., Cristina S., Sandrini G., Nappi G. (2000). Botulinum Toxin Treatment for Functional Disability Induced by Essential Tremor. Neurol. Sci..

[28] Kreisler A., Bouchain B., Defebvre L., Krystkowiak P. (2019). Treatment with Botulinum Neurotoxin Improves Activities of Daily Living and Quality of Life in Patients with Upper Limb Tremor. Tremor Hyperkinetic Mov..

[29] Samotus O., Rahimi F., Lee J., Jog M. (2016). Functional Ability Improved in Essential Tremor by IncobotulinumtoxinA Injections Using Kinematically Determined Biomechanical Patterns—A New Future. PLoS ONE.

[30] Samotus O., Kumar N., Rizek P., Jog M. (2018). Botulinum Toxin Type A Injections as Monotherapy for Upper Limb Essential Tremor Using Kinematics. Can. J. Neurol. Sci. J. Can. Sci. Neurol..

[31] Samotus O., Lee J., Jog M. (2019). Personalized Bilateral Upper Limb Essential Tremor Therapy with Botulinum Toxin Using Kinematics. Toxins.

[32] Samotus O., Mahdi Y., Jog M. (2022). Real-World Longitudinal Experience of Botulinum Toxin Therapy for Parkinson and Essential Tremor. Toxins.

[33] Salazar G., Caballero I. (2025). Ultrasound-Guided Botulinum Toxin Infiltrations in Essential Tremor Patients: A 36-Week Follow Up. Tremor Hyperkinetic Mov. N. Y..

[34] Varghese A., Berry D.S., Ghanem A., Hernandez N.C., Louis E.D. (2024). Patient-Reported Treatment Satisfaction in Essential Tremor: Levels of Satisfaction and Predictors of Satisfaction. Ther. Adv. Neurol. Disord..

[35] Ferreira J.J., Mestre T.A., Lyons K.E., Benito-León J., Tan E.-K., Abbruzzese G., Hallett M., Haubenberger D., Elble R., Deuschl G. (2019). MDS Evidence-Based Review of Treatments for Essential Tremor. Mov. Disord. Off. J. Mov. Disord. Soc..

[36] Pahwa R., Busenbark K., Swanson-Hyland E.F., Dubinsky R.M., Hubble J.P., Gray C., Koller W.C. (1995). Botulinum Toxin Treatment of Essential Head Tremor. Neurology.

[37] Zheng X., Wei W., Liu P., Wu C., Lu L., Tang C. (2020). Botulinum Toxin Type A for Hand Tremor: A Meta-Analysis of Randomised Controlled Trials. Neurol. Neurochir. Pol..

[38] Liao Y.-H., Hong C.-T., Huang T.-W. (2022). Botulinum Toxin for Essential Tremor and Hands Tremor in the Neurological Diseases: A Meta-Analysis of Randomized Controlled Trials. Toxins.

[39] Louis E.D. (2025). Cranial Tremors in Essential Tremor: Prevalence, Anatomic Distribution and Predictors of Occurrence. Mov. Disord. Clin. Pract..

[40] Bellows S.T., Jankovic J. (2021). Phenotypic Features of Isolated Essential Tremor, Essential Tremor Plus, and Essential Tremor-Parkinson’s Disease in a Movement Disorders Clinic. Tremor Hyperkinetic Mov. N. Y..

[41] Beylergil S.B., Mukunda K.N., Elkasaby M., Perlmutter J.S., Factor S., Bäumer T., Feurestein J., Shelton E., Bellows S., Jankovic J. (2024). Tremor in Cervical Dystonia. Dystonia.

[42] Pandey S. (2024). More Evidence for Botulinum Toxin in Isolated or Essential Head Tremor. Mov. Disord. Clin. Pract..

[43] Marques A., Pereira B., Simonetta-Moreau M., Castelnovo G., Verdal M.D., Fluchère F., Laurencin C., Degos B., Tir M., Kreisler A. (2023). Trial of Botulinum Toxin for Isolated or Essential Head Tremor. N. Engl. J. Med..

[44] Van Der Walt A., Sung S., Spelman T., Marriott M., Kolbe S., Mitchell P., Evans A., Butzkueven H. (2012). A Double-Blind, Randomized, Controlled Study of Botulinum Toxin Type A in MS-Related Tremor. Neurology.

[45] Boonstra F.M.C., Evans A., Noffs G., Perera T., Jokubaitis V., Stankovich J., Vogel A.P., Moffat B.A., Butzkueven H., Kolbe S.C. (2020). OnabotulinumtoxinA Treatment for MS-Tremor Modifies fMRI Tremor Response in Central Sensory-Motor Integration Areas. Mult. Scler. Relat. Disord..

[46] Nagaratnam S.A., Wilson D., Chiang H.-L., Chang F.C.F., Qiu J., Silsby M., Fois A.F., Martin A., Mahant N., Fung V.S.C. (2025). A Randomized, Double-Blind, Placebo Controlled, Crossover Trial of incobotulinumtoxinA Treatment for Upper Limb Tremor. Mov. Disord. Clin. Pract..

[47] Rajan R., Srivastava A.K., Anandapadmanabhan R., Saini A., Upadhyay A., Gupta A., Vishnu V.Y., Pandit A.K., Vibha D., Singh M.B. (2021). Assessment of Botulinum Neurotoxin Injection for Dystonic Hand Tremor: A Randomized Clinical Trial. JAMA Neurol..

[48] Wissel J., Masuhr F., Schelosky L., Ebersbach G., Poewe W. (1997). Quantitative Assessment of Botulinum Toxin Treatment in 43 Patients with Head Tremor. Mov. Disord..

[49] Bessemer R.A., Jog M. (2024). Botulinum Toxin Injections to the Obliquus Capitis Inferioris Muscle for Dynamic Cervical Dystonia Improves Subjective Patient Outcomes. Toxins.

[50] Lester R.A., Barkmeier-Kraemer J., Story B.H. (2013). Physiologic and Acoustic Patterns of Essential Vocal Tremor. J. Voice.

[51] Louis E.D. (2014). Twelve Clinical Pearls to Help Distinguish Essential Tremor from Other Tremors. Expert Rev. Neurother..

[52] Richards A.L. (2017). Vocal Tremor: Where Are We At?. Curr. Opin. Otolaryngol. Head Neck Surg..

[53] Warrick P., Dromey C., Irish J.C., Durkin L., Pakiam A., Lang A. (2000). Botulinum Toxin for Essential Tremor of the Voice with Multiple Anatomical Sites of Tremor: A Crossover Design Study of Unilateral Versus Bilateral Injection. Laryngoscope.

[54] Adler C.H., Bansberg S.F., Hentz J.G., Ramig L.O., Buder E.H., Witt K., Edwards B.W., Krein-Jones K., Caviness J.N. (2004). Botulinum Toxin Type A for Treating Voice Tremor. Arch. Neurol..

[55] Justicz N., Hapner E.R., Josephs J.S., Boone B.C., Jinnah H.A., Johns M.M. (2016). Comparative Effectiveness of Propranolol and Botulinum for the Treatment of Essential Voice Tremor. Laryngoscope.

[56] Guglielmino G., de Moraes B.T., Villanova L.C., Padovani M., Biase N.G.D. (2018). Comparison of Botulinum Toxin and Propranolol for Essential and Dystonic Vocal Tremors. Clinics.

[57] Hertegård S., Granqvist S., Lindestad P.-Å. (2000). Botulinum Toxin Injections for Essential Voice Tremor. Ann. Otol. Rhinol. Laryngol..

[58] Gurey L.E., Sinclair C.F., Blitzer A. (2013). A New Paradigm for the Management of Essential Vocal Tremor with Botulinum Toxin. Laryngoscope.

[59] Stone A., Powell M.E., Hamers K., Fletcher K.C., Francis D.O., Courey M.S., Netterville J.L., Garrett C.G. (2022). Optimizing Botox Regimens in Patients with Adductor Spasmodic Dysphonia and Essential Tremor of Voice: A 31-year Experience. Laryngoscope Investig. Otolaryngol..

[60] Khoury S., Randall D.R. (2024). Treatment of Essential Vocal Tremor: A Scoping Review of Evidence-Based Therapeutic Modalities. J. Voice Off. J. Voice Found..

[61] Anandan C., Jankovic J. (2024). Botulinum Toxin Treatment in Parkinsonism. J. Neurol. Sci..

[62] Rahimi F., Bee C., Debicki D., Roberts A.C., Bapat P., Jog M. (2013). Effectiveness of BoNT A in Parkinson’s Disease Upper Limb Tremor Management. Can. J. Neurol. Sci. J. Can. Sci. Neurol..

[63] Rahimi F., Samotus O., Lee J., Jog M. (2015). Effective Management of Upper Limb Parkinsonian Tremor by IncobotulinumtoxinA Injections Using Sensor-Based Biomechanical Patterns. Tremor Hyperkinetic Mov..

[64] Samotus O., Lee J., Jog M. (2020). Standardized Algorithm for Muscle Selection and Dosing of Botulinum Toxin for Parkinson Tremor Using Kinematic Analysis. Ther. Adv. Neurol. Disord..

[65] Schneider S.A., Edwards M.J., Cordivari C., Macleod W.N., Bhatia K.P. (2006). Botulinum Toxin A May Be Efficacious as Treatment for Jaw Tremor in Parkinson’s Disease. Mov. Disord. Off. J. Mov. Disord. Soc..

[66] Eslamian F., Dolatkhah N., Fallah L., Jahanjoo F., Toopchizadeh V., Talebi M. (2023). Effectiveness of Botulinum Toxin on Hand Tremor Intensity and Upper Limb Function in Patients with Parkinson’s Disease: Results of a Systematic Review. Tremor Hyperkinetic Mov. N. Y..

[67] Gao F., Zhao J., Wang C., Hu L., Gao C. (2024). The Effectiveness and Safety of Botulinum Toxin Injections for Managing Motor Disorders of Patients with Parkinson’s Disease: A Meta-Analysis of Randomized Controlled Trials. Basic Clin. Pharmacol. Toxicol..

[68] Bruno V., Freitas M.E., Mancini D., Lui J.P., Miyasaki J., Fox S.H. (2018). Botulinum Toxin Type A for Pain in Advanced Parkinson’s Disease. Can. J. Neurol. Sci. J. Can. Sci. Neurol..

[69] Mehanna R., Jankovic J. (2013). Movement Disorders in Multiple Sclerosis and Other Demyelinating Diseases. J. Neurol. Sci..

[70] Clarke C.E. (1997). Botulinum Toxin Type A in Cerebellar Tremor Caused by Multiple Sclerosis. Eur. J. Neurol..

[71] Gonul Oner O., Erturk Cetin O., Serkan D. (2024). A Retrospective Study on Botulinum Toxin Injection in Patients With Multiple Sclerosis Related Tremor: A Treatment Option Worth Trying. Clin. Neuropharmacol..

[72] Boghen D., Flanders M. (1993). Effectiveness of Botulinum Toxin in the Treatment of Spasmodic Torticollis. Eur. Neurol..

[73] Borodic G.E., Mills L., Joseph M. (1991). Botulinum A Toxin for the Treatment of Adult-Onset Spasmodic Torticollis. Plast. Reconstr. Surg..

[74] Fasano A., Bove F., Lang A.E. (2014). The Treatment of Dystonic Tremor: A Systematic Review. J. Neurol. Neurosurg. Psychiatry.

[75] González-Herrero B., Di Vico I.A., Pereira E., Edwards M., Morgante F. (2023). Treatment of Dystonic Tremor of the Upper Limbs: A Single-Center Retrospective Study. J. Clin. Med..

[76] Lee A., Furuya S., Altenmüller E. (2014). Epidemiology and Treatment of 23 Musicians with Task Specific Tremor. J. Clin. Mov. Disord..

[77] Papapetropoulos S., Singer C. (2006). Treatment of Primary Writing Tremor With Botulinum Toxin Type A Injections: Report of a Case Series. Clin. Neuropharmacol..

[78] Dreissen Y.E.M., Dijk J.M., Gelauff J.M., Zoons E., van Poppelen D., Contarino M.F., Zutt R., Post B., Munts A.G., Speelman J.D. (2019). Botulinum Neurotoxin Treatment in Jerky and Tremulous Functional Movement Disorders: A Double-Blind, Randomised Placebo-Controlled Trial with an Open-Label Extension. J. Neurol. Neurosurg. Psychiatry.

[79] Garcin B., Mesrati F., Hubsch C., Mauras T., Iliescu I., Naccache L., Vidailhet M., Roze E., Degos B. (2017). Impact of Transcranial Magnetic Stimulation on Functional Movement Disorders: Cortical Modulation or a Behavioral Effect?. Front. Neurol..

[80] Grigoriu A.-I., Dinomais M., Rémy-Néris O., Brochard S. (2015). Impact of Injection-Guiding Techniques on the Effectiveness of Botulinum Toxin for the Treatment of Focal Spasticity and Dystonia: A Systematic Review. Arch. Phys. Med. Rehabil..

[81] Molloy F.M., Shill H.A., Kaelin–Lang A., Karp B.I. (2002). Accuracy of Muscle Localization without EMG: Implications for Treatment of Limb Dystonia. Neurology.

[82] Picelli A., Roncari L., Baldessarelli S., Berto G., Lobba D., Santamato A., Fiore P., Smania N. (2014). Accuracy of Botulinum Toxin Type A Injection into the Forearm Muscles of Chronic Stroke Patients with Spastic Flexed Wrist and Clenched Fist: Manual Needle Placement Evaluated Using Ultrasonography. J. Rehabil. Med..

[83] Sitburana O., Wu L.J.C., Sheffield J.K., Davidson A., Jankovic J. (2009). Motor Overflow and Mirror Dystonia. Park. Relat. Disord..

[84] Motavasseli D., Delorme C., Bayle N., Gracies J.-M., Roze E., Baude M. (2024). Use of Botulinum Toxin in Upper-Limb Tremor: Systematic Review and Perspectives. Toxins.

[85] Alter K.E., Karp B.I. (2017). Ultrasound Guidance for Botulinum Neurotoxin Chemodenervation Procedures. Toxins.

[86] Hong J.S., Sathe G.G., Niyonkuru C., Munin M.C. (2012). Elimination of Dysphagia Using Ultrasound Guidance for Botulinum Toxin Injections in Cervical Dystonia. Muscle Nerve.

[87] Walter U., Dressler D. (2014). Ultrasound-Guided Botulinum Toxin Injections in Neurology: Technique, Indications and Future Perspectives. Expert Rev. Neurother..

[88] Comella C.L., Buchman A.S., Tanner C.M., Brown-Toms N.C., Goetz C.G. (1992). Botulinum Toxin Injection for Spasmodic Torticollis: Increased Magnitude of Benefit with Electromyographic Assistance. Neurology.

[89] Ploumis A., Varvarousis D., Konitsiotis S., Beris A. (2014). Effectiveness of Botulinum Toxin Injection with and without Needle Electromyographic Guidance for the Treatment of Spasticity in Hemiplegic Patients: A Randomized Controlled Trial. Disabil. Rehabil..

[90] Werdelin L., Dalager T., Fuglsang-Frederiksen A., Regeur L., Karlsborg M., Korbo L., Munck O., Winge K. (2011). The Utility of EMG Interference Pattern Analysis in Botulinum Toxin Treatment of Torticollis: A Randomised, Controlled and Blinded Study. Clin. Neurophysiol. Off. J. Int. Fed. Clin. Neurophysiol..

[91] Picelli A., Lobba D., Midiri A., Prandi P., Melotti C., Baldessarelli S., Smania N. (2014). Botulinum Toxin Injection into the Forearm Muscles for Wrist and Fingers Spastic Overactivity in Adults with Chronic Stroke: A Randomized Controlled Trial Comparing Three Injection Techniques. Clin. Rehabil..

[92] Xu K., Yan T., Mai J. (2009). A Randomized Controlled Trial to Compare Two Botulinum Toxin Injection Techniques on the Functional Improvement of the Leg of Children with Cerebral Palsy. Clin. Rehabil..

[93] Picelli A., Tamburin S., Bonetti P., Fontana C., Barausse M., Dambruoso F., Gajofatto F., Santilli V., Smania N. (2012). Botulinum Toxin Type A Injection into the Gastrocnemius Muscle for Spastic Equinus in Adults with Stroke: A Randomized Controlled Trial Comparing Manual Needle Placement, Electrical Stimulation and Ultrasonography-Guided Injection Techniques. Am. J. Phys. Med. Rehabil..

[94] Py A.-G., Zein Addeen G., Perrier Y., Carlier R.-Y., Picard A. (2009). Evaluation of the Effectiveness of Botulinum Toxin Injections in the Lower Limb Muscles of Children with Cerebral Palsy. Preliminary Prospective Study of the Advantages of Ultrasound Guidance. Ann. Phys. Rehabil. Med..

[95] Chan A.K., Finlayson H., Mills P.B. (2017). Does the Method of Botulinum Neurotoxin Injection for Limb Spasticity Affect Outcomes? A Systematic Review. Clin. Rehabil..

[96] Park S.-H., Shin J.-H. (2024). Ultrasonography for Assessment and Intervention With Botulinum Toxin Injection for Tremors. Ann. Rehabil. Med..

[97] Wissel J., Ward A.B., Erztgaard P., Bensmail D., Hecht M.J., Lejeune T.M., Schnider P., Altavista M.C., Cavazza S., Deltombe T. (2009). European Consensus Table on the Use of Botulinum Toxin Type A in Adult Spasticity. J. Rehabil. Med..

[98] Simpson D.M., Hallett M., Ashman E.J., Comella C.L., Green M.W., Gronseth G.S., Armstrong M.J., Gloss D., Potrebic S., Jankovic J. (2016). Practice Guideline Update Summary: Botulinum Neurotoxin for the Treatment of Blepharospasm, Cervical Dystonia, Adult Spasticity, and Headache: Report of the Guideline Development Subcommittee of the American Academy of Neurology. Neurology.

[99] Albanese A., Abbruzzese G., Dressler D., Duzynski W., Khatkova S., Marti M.J., Mir P., Montecucco C., Moro E., Pinter M. (2015). Practical Guidance for CD Management Involving Treatment of Botulinum Toxin: A Consensus Statement. J. Neurol..

[100] Pigg A.C., Thompson-Westra J., Mente K., Maurer C.W., Haubenberger D., Hallett M., Charles S.K. (2020). Distribution of Tremor among the Major Degrees of Freedom of the Upper Limb in Subjects with Essential Tremor. Clin. Neurophysiol. Off. J. Int. Fed. Clin. Neurophysiol..

[101] Mittal S.O., Pandey S. (2022). Botulinum Toxin for the Treatment of Tremor. J. Neurol. Sci..

[102] Friedrich M.U., Roenn A.-J., Palmisano C., Alty J., Paschen S., Deuschl G., Ip C.W., Volkmann J., Muthuraman M., Peach R. (2024). Validation and Application of Computer Vision Algorithms for Video-Based Tremor Analysis. NPJ Digit. Med..

[103] Wolke R., Welzel J., Maetzler W., Deuschl G., Becktepe J. (2025). Validity of Tremor Analysis Using Smartphone Compatible Computer Vision Frameworks. Sci. Rep..

